# Supraspinatus Intramuscular Calcified Hematoma or Necrosis Associated with Tendon Tear

**DOI:** 10.1155/2015/496313

**Published:** 2015-08-26

**Authors:** Alexandre Lädermann, Muriel Genevay, Sophie Abrassart, Adrien Jean-Pierre Schwitzguébel

**Affiliations:** ^1^Division of Orthopaedics and Trauma Surgery, La Tour Hospital, rue J.-D. Maillard 3, 1217 Meyrin, Switzerland; ^2^Faculty of Medicine, University of Geneva, rue Michel-Servet 1, 1211 Geneva, Switzerland; ^3^Division of Orthopaedics and Trauma Surgery, Department of Surgery, Geneva University Hospitals, rue Gabrielle-Perret-Gentil 4, 1211 Geneva, Switzerland; ^4^Laboratoire Dianapath, rue de la Colline 10, 1205 Geneva, Switzerland

## Abstract

*Introduction*. Rotator cuff intramuscular calcification is a rare condition usually caused by heterotopic ossification and myositis ossificans. *Case Presentation*. We describe a patient with voluminous calcified mass entrapped in supraspinatus muscle associated with corresponding tendon tear. Histological examination corresponded to a calcified hematoma or necrosis. Patient was surgically managed with open excision of the calcified hematoma and rotator cuff arthroscopic repair. At 6 months, supraspinatus muscle was healed, and functional outcome was good. *Discussion and Conclusion*. We hypothesized that supraspinatus intramuscular calcified hematoma was responsible for mechanical stress on the tendon. This association has never been described.

## 1. Introduction

Rotator cuff ossification is a rare condition, poorly documented in the literature, except in case of heterotopic ossification, a well-known complication of severe acute neurologic disease [1] and surgery [2]. Enclosed rotator cuff muscles ossification is an exceptional condition [3] that might be caused by secondary calcified muscle hematoma or necrosis [4], myositis ossificans [3, 5–8], and congenital diseases like progressive fibrodysplasia ossificans that has a prevalence of 1/2,000,000 [9]. Calcified intramuscular hematoma or necrosis is often deep seated slowly enlarging lesions that may be discovered as long as 20 years after the initial traumatic event. Histologically they are centered by blood clot, fibrin, and prominent amorphous debris. Dystrophic calcifications and scar tissue may also be seen [10]. Myositis ossificans is defined as a benign solitary self-limiting ossifying soft tissue mass that typically occurs in skeletal muscle [11] and is described as commonly associated with trauma, reported by a small number of case reports [6, 12–14]. It has been proposed that when no clear traumatic mechanism is reported, repetitive microtraumatic injuries, ischemia, and chronic inflammation could be myositis ossificans etiological factors [15]. In this way, repetitive microtrauma in shoulder area in soldiers has been described as an etiologic mechanism of myositis ossificans [16]. Histologically, myositis ossificans can present different maturation stages. It has been postulated that intramuscular hematoma is the initial lesion contributing to myositis ossificans and that lesions are considered as fully calcified or “mature,” 5 months after initial trauma [17, 18].

## 2. Case Presentation

A 75-year-right-hand-dominant woman presented with right shoulder pain that evolved over 5 years. She had history of right supraspinatus muscle trauma 30 years ago, but she had not noticed any traumatic, infectious, or iatrogenic event on the right shoulder on those 5 last years. Although she had done well previously, in recent months she had worsening symptoms that was affecting her quality of life, with a visual analog scale score for pain of 9 on a scale of 10, a Single Assessment Numeric Evaluation score of 40 [19], and a Constant Shoulder Score of 64 [20]. Symptoms were refractory to pain killers and physical therapy. No limitation in shoulder range of motion was observed.

**Figure 1:**
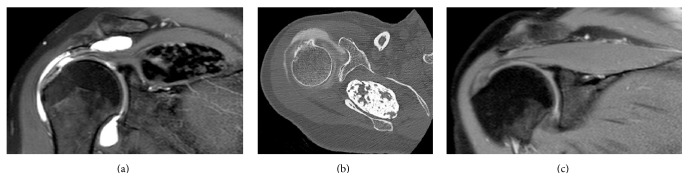
Radiological evaluation of supraspinatus muscle and tendon. MRA showed a calcified mass, as well as a supraspinatus tendon thinning (a). Preoperative CT was performed for a better definition of the volume of the well-delimited calcified mass into supraspinatus muscle (b). Five years ago, a supraspinatus muscle MRI excluded intramuscular mass on this time (c).

MRI showed a thinned supraspinatus tendon (Figure 1(a)). A complementary CT scan showed a well-delimited voluminous supraspinatus muscle calcification (Figure 1(b)), as well as an infra-acromial spur. Five years ago, a prior MRI excluded any supraspinatus muscle mass (Figure 1(c)). At this point and with described elements, we suspected the diagnosis of supraspinatus intramuscular calcification.

**Figure 2:**
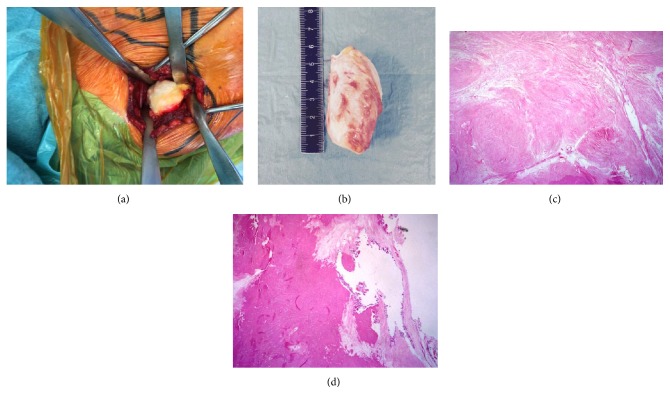
In situ, macroscopic, and histologic aspects of calcified mass. In situ (a) and postextraction (b) macroscopic aspects of calcified mass showed a 7 × 4 × 4 cm well-delimited whitish solid tumor. Histologic evaluation ((c) and (d)) showed a highly hyalinized collagenous scar tissue devoid of any inflammatory cells, with some dystrophic calcifications at the periphery of the lesion (d).

Surgery was performed in order to remove the calcified supraspinatus mass. A small type A [21] rotator cuff lesion was also repaired under arthroscopy. First, tension on supraspinatus tendon was reduced by extracting calcific mass (Figure 2(a)). Next, an open approach between the trapezius and supraspinatus muscles was performed. A cleavage plane between calcific mass and supraspinatus muscle was well-delineated. Therefore, excision margins were not necessary. Then, arthroscopic procedure was performed to repair supraspinatus and superior subscapularis tendons with side to side point. Lateral acromioplasty, as well as bursectomy, was also performed.

Macroscopically, the lesion was a well-defined 7 × 4 × 4 cm whitish fusiform lesion (Figure 2(b)). Histologic examination revealed a highly hyalinized collagenous stroma almost acellular. Few scattered capillary vessels could be seen. The thickness of their wall was normal and they were surrounded by few plasma cells. Less than 1 mm foci of fat tissue and lamellar bone formation could also be seen. Dystrophic calcifications were observed at the periphery of the lesion. There was neither inflammatory nor giant cell reaction around it. A Congo-red coloration has been performed to exclude an amyloidoma that was negative (Figures 2(c) and 2(d)). Diagnosis of calcified hematoma or necrosis was confirmed.

**Figure 3:**
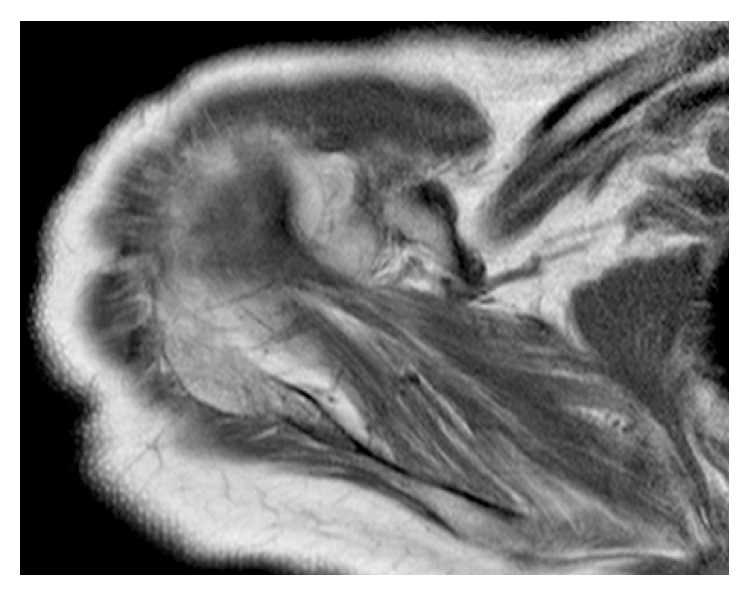
Supraspinatus muscle six months after surgery radiologic evaluation. MRA showed a good healing of the supraspinatus muscle. However, a Goutallier stage 2 fatty infiltration remains.

Follow-up included 2 weeks of immobilization in 30° abduction and then passive motion in all directions. Six months after surgery, the patient was satisfied of her condition, with a complete shoulder range of motion recovery, a visual analog scale score for pain of 4 on a scale of 10, a Single Assessment Numeric Evaluation score of 40 [19], and a Constant Shoulder Score of 66 [20]. MRA showed a supraspinatus muscle with Goutallier stage 2 fatty infiltration [22], as well as the absence of “fish backbone sign” [23] or recurrence of calcified mass (Figure 3).

## 3. Discussion

Circumscribed ossifications into rotator cuff muscles are rare conditions. We only found myositis ossificans case reports in the English literature [3, 5–8]. However, these conditions are poorly described in the literature. To our knowledge, voluminous calcified hematoma or necrosis into the rotator cuff has never been described. We suppose that calcified hematoma or necrosis might be confused with myositis ossificans in absence of pathological examination or that publications concerning those entities considered as “basic” or “simple” might be omitted. Moreover, intramuscular hematoma might be on the origin of myositis ossificans or calcified hematoma. In this way, myositis ossificans is discussed in a large-scale review of the literature on the subject by King [4], but not calcified hematoma.

The patient in question had a typical clinical presentation of chronic superior rotator cuff tendinopathy evolving since 5 years; the symptoms began before the calcific mass was present. It is therefore possible that calcific mass appearance decompensated a chronic supraspinatus tendon tear by increasing mechanical traction on supraspinatus muscle. The effectiveness of conservative management for that supraspinatus tendon tear was therefore compromised. For this reason, surgical management of both calcified mass and supraspinatus tendon tear was proposed, even if calcific mass was not painful and could have been subject to conservative management, especially with NSAIDs [3, 24]. Note that when therapeutic management was set up, the presumptive diagnosis was a myositis ossificans.

Typically, stress on the supraspinatus arises in the context of a subacromial impingement [25]. This case report illustrates a rare case of intramuscular mechanical stress factor on the supraspinatus tendon. We could find only one case report of supraspinatus tendon surgery that included alleviation of a muscular stress factor, dating from 1992 [8]. Clinical outcome was good.

For the first time in arthroscopic rotator cuff surgery, to the best of our knowledge, we cured a muscular factor that might decompensate chronic rotator cuff pathology. This association between chronic supraspinatus tendinopathy and supraspinatus muscle calcific mass was managed by association muscular mechanical stress alleviation and arthroscopic rotator cuff repair, with a good muscle healing and satisfying functional outcome at 6-month-postsurgery checkup.
